# Association of malnutrition with renal dysfunction and clinical outcome in patients with heart failure

**DOI:** 10.1038/s41598-022-20985-z

**Published:** 2022-10-05

**Authors:** Yoichiro Otaki, Tetsu Watanabe, Mari Shimizu, Shingo Tachibana, Junya Sato, Yuta Kobayashi, Yuji Saito, Tomonori Aono, Harutoshi Tamura, Shigehiko Kato, Satoshi Nishiyama, Hiroki Takahashi, Takanori Arimoto, Masafumi Watanabe

**Affiliations:** 1 Department of Cardiology, Pulmonology, and Nephrology, Yamagata University School of Medicine, 2-2-2 Iida-Nishi, Yamagata, 990-9585 Japan; 2 Faculty of Medicine, Yamagata University School of Medicine, Yamagata, Japan

**Keywords:** Cardiology, Nephrology, Risk factors

## Abstract

Malnutrition, glomerular damage (GD), and renal tubular damage (RTD) are common morbidities associated with poor clinical outcomes in heart failure (HF) patients. However, the association between malnutrition and renal dysfunction and its impact on clinical outcomes in HF patients have not yet been fully elucidated. We assessed the nutritional status and renal function of 1061 consecutive HF patients. Malnutrition, GD, and RTD were defined as a controlling nutritional status (CONUT) score of ≥ 5, reduced eGFR or microalbuminuria, and levels of *N*-acetyl-beta-d-glucosamidase of > 14.2 U/gCr according to previous reports, respectively. Patients with RTD had a higher CONUT score and a lower prognostic nutritional index and geriatric nutritional risk index than those without. Multivariate logistic analysis demonstrated that RTD, but not GD, was significantly associated with malnutrition. There were 360 cardiac events during the median follow-up period of 688 days. Multivariate Cox proportional hazard regression analysis demonstrated that comorbid malnutrition and renal dysfunction, rather than simple malnutrition, were significantly associated with cardiac events in HF patients. We found a close relationship between malnutrition and renal dysfunction in HF patients. Comorbid malnutrition and renal dysfunction were risk factors for cardiac events in HF patients, suggesting the importance of managing and treating these.

## Introduction

Despite advances in medicine, heart failure (HF) remains an increasing public health problem associated with high morbidity and mortality^1^. Malnutrition has been reported as common and associated with poor clinical outcomes in patients with HF^2^. Objective nutritional assessments have been recommended in patients with HF, such as the prognostic nutritional index (PNI), geriatric nutritional risk index (GNRI) and controlling nutritional status (CONUT) score^3–5^. The CONUT score, comprised serum albumin, lymphocyte count, and total cholesterol values, is a useful tool to assess objective nutritional status in patients with cardiovascular disease^6–8^. Recent reports indicate that the major causes of disease-related malnutrition are HF, infectious disease, and chronic kidney disease (CKD)^9^. Although the major underlying mechanism of malnutrition is considered to be inflammation, other factors predisposing to malnutrition in patients with HF have not yet been fully elucidated.

Renal dysfunction is a well-established risk factor for poor clinical outcomes in patients with HF, as comorbid HF and renal dysfunction comprise a vicious cycle^10^. Overall renal function is assessed by cause (C) glomerular filtration rate (G) albuminuria (A) staging according to the Kidney Disease: Improving Global Outcome (K/DIGO) guidelines^11^. HF-induced renal dysfunction includes both glomerular damage (GD) and renal tubular damage (RTD). Previous reports have demonstrated that glomerular filtration rate, albuminuria, and tubulointerstitial damage are all associated with poor prognosis in patients with HF^12,13^. Accumulating evidence has demonstrated a close relationship between malnutrition and CKD^14^. However, it remains unclear whether renal dysfunction is related to malnutrition in patients with HF.

As GD and RTD are also common in patients with HF, we hypothesized that there are many patients with comorbid malnutrition and renal dysfunction in patients with HF. The purposes of the present study were to (1) examine whether renal dysfunction was related to malnutrition in patients with HF and (2) examine the impact of comorbid malnutrition and renal dysfunction on cardiac events in patients with HF.

## Results

### Baseline characteristics of all patients with HF and comparisons of clinical characteristics of patients with and without malnutrition

The baseline characteristics of the patients are presented in Table 1. There were 407, 377, and 277 patients in NYHA functional classes II, III, and IV, respectively. Hypertension, diabetes mellitus, and dyslipidemia were identified in 846 (80%), 402 (38%), and 631 (59%) patients, respectively. The etiology of HF was ischemic heart disease in 253 (24%) patients, dilated cardiomyopathy in 159 (15%) patients, and other conditions in the remaining 649 (61%) patients. Malnutrition, defined as a high CONUT score (≥ 5), was identified in 429 (40%) patients with HF. The mean CONUT score, PNI, and GNRI were 4.0, 40.8, and 94, respectively. The mean serum albumin, lymphocyte count, and total cholesterol were 3.4 g/dL, 1,400/mm^3^, and 160 mg/dL, respectively. The mean eGFR, median UACR, and median NAG were 61 mL/min/1.73 m^2^, 43 (15–142) mg/gCr, and 11.6 (7–20) U/gCr, respectively. GD and RTD were identified in 804 (75%) and 412 (39%) patients, respectively. The mean left ventricular ejection fraction and septal E/e′ ratio were 48.8% and 16.5, respectively.

**Table 1:** Comparisons of baseline clinical characteristics between patients with and without malnutrition defined by high CONUT score.

Variables	All patients n = 1061	Malnutrition (−) n = 632	Malnutrition (+) n = 429	P value
Age	72 ± 13	69 ± 14	76 ± 12	< 0.0001
Male/female, n	640/421	386/246	254/175	0.5417
Etiology of heart failure IHD/DCM/Others	253/159/649	125/117/390	128/42/259	< 0.0001
NYHA functional class II/III/IV, n	407/377/277	311/191/130	96/186/147	< 0.0001
Hypertension, n (%)	846 (80%)	503 (80%)	343 (80%)	0.8846
Diabetes mellitus, n (%)	402 (38%)	225 (36%)	177 (41%)	0.0627
Dyslipidemia, n (%)	631 (59%)	375 (59%)	256 (60%)	0.9123
Atrial fibrillation, n (%)	436 (41%)	243 (38%)	193 (45%)	0.0338
ICD/CRT-D implantation, n (%)	35 (3.3%)	27 (4.3%)	8 (1.9%)	0.0312
Systolic blood pressure, mmHg	135 ± 34	136 ± 35	134 ± 33	0.4358
Diastolic blood pressure, mmHg	78 ± 22	79 ± 21	77 ± 22	0.0972
Heart rate, beat per minute	90 ± 28	89 ± 29	90 ± 28	0.4517
**Nutritional status**				
Serum albumin, g/dL	3.4 ± 0.4	3.7 ± 0.4	2.9 ± 0.4	< 0.0001
Lymphocyte count, mm^3^	1,400 ± 568	1,644 ± 630	1,041 ± 460	< 0.0001
Total cholesterol, mg/dL	160 ± 37	175 ± 37	139 ± 36	< 0.0001
CONUT score	4.0 ± 1.5	2.0 ± 1.4	6.8 ± 1.8	< 0.0001
PNI	40.8 ± 5.1	45.3 ± 5.5	34.1 ± 4.5	< 0.0001
GNRI	94 ± 10	99 ± 10	85 ± 10	< 0.0001
**Renal function**				
eGFR, ml/min/1.73 m^2^	61 ± 29	64 ± 25	57 ± 35	0.0002
UACR, mg/gCr	43 (15–142)	28 (9–87)	76 (28–259)	< 0.0001
NAG, U/gCr	11.6 (7–20)	9.3 (5.9–15.1)	15.8 (9.8–25.5)	< 0.0001
GD, n (%)	804 (75%)	426 (67%)	378 (88%)	< 0.0001
RTD, n (%)	412 (39%)	172 (27%)	240 (56%)	< 0.0001
**Biochemical data**				
BNP, pg/mL	470 (187–882)	368 (141–726)	612 (304–1121)	< 0.0001
hsCRP, mg/dL	0.457 (0.104–1.020)	0.218 (0.067–0.849)	1.020 (0.326–1.020)	< 0.0001
Hemoglobin, g/dL	12.0 ± 2.3	12.9 ± 2.3	10.8 ± 2.5	< 0.0001
Sodium, mEq/L	140.4 ± 3.6	140.8 ± 3.3	139.7 ± 4.1	< 0.0001
**Echocardiogram**				
LVEF, %	48.8 ± 17.7	48.5 ± 18.4	49.4 ± 16.7	0.4295
LVEDD, mm	54 ± 10	55 ± 10	53 ± 10	0.0472
E/e’ ratio	16.5 ± 9.3	16.1 ± 9.4	17.1 ± 9.3	0.1539
**Medicines**				
ACEIs, ARBs, n (%)	665 (63%)	398 (63%)	267 (62%)	0.8076
β-blockers, n (%)	760 (72%)	461 (73%)	299 (70%)	0.2506
Mineralocorticoid receptor antagonists, n (%)	342 (32%)	195 (31%)	147 (34%)	0.2440
Diuretics, n (%)	678 (64%)	366 (58%)	312 (73%)	< 0.0001

Data are expressed as mean ± SD, number (percentage), or median (interquartile range).

*ACEIs* angiotensin-converting enzyme inhibitors, *ARBs* angiotensin II receptor blockers, *BNP* brain natriuretic peptide, *CONUT* controlling nutritional status, *CRT-D* cardiac resynchronization therapy defibrillator, *DCM* dilated cardiomyopathy, *E/e′ ratio* the ratio of the mitral inflow E wave to the tissue Doppler e’ wave, *eGFR* estimated glomerular filtration rate, *GD* glomerular damage, *GNRI* geriatric nutritional risk index, *ICD* implantable cardioverter defibrillator, *IHD* ischemic heart disease, *LVEDD* left ventricular end diastolic dimension, *LVEF* left ventricular ejection fraction, *hsCRP* high sensitivity C-reactive protein, *NAG* *N*-acetyl-beta-d-glucosamidase, *NYHA* New York Heart Association, *PNI* prognostic nutritional status, *RTD* renal tubular damage, *UACR* urinary microalbumin-creatinine ratio.

As shown in Table 1, patients with malnutrition were older, had a more severe NYHA functional class, had lower levels of eGFR and left ventricular end diastolic dimension, and higher levels of UACR, NAG, BNP, and hsCRP than those without malnutrition. The prevalence rates of atrial fibrillation, GD, and RTD were higher in patients with malnutrition than in those without it.

### Association between malnutrition and renal dysfunction in HF

**Figure 1:**
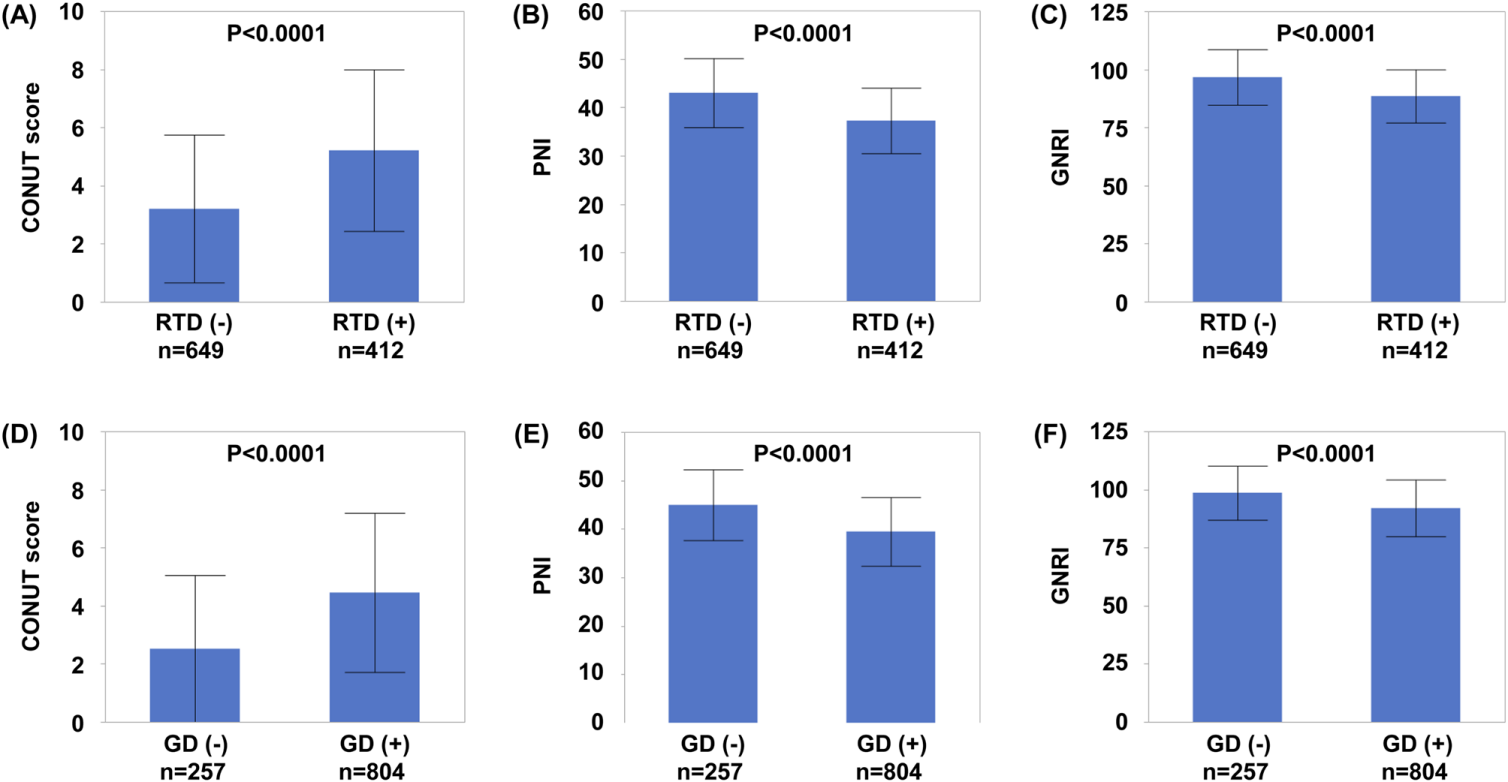
Association between malnutrition and renal dysfunction; (**A–C**) The association of RTD with CONUT score, PNI, and GNRI; (**D–F**) The association of GD with CONUT score, PNI, and GNRI. *CONUT* controlling nutritional status, *GD* glomerular damage, *GNRI* geriatric nutritional risk index, *PNI* prognostic nutritional index, *RTD* renal tubular damage.

As shown in Fig. 1, patients with RTD had higher CONUT scores and lower PNI and GNRI than those without RTD in all HF patients. Moreover, patients with GD had higher CONUT score and lower PNI and GNRI than those without GD in all HF patients.

**Figure 2:**
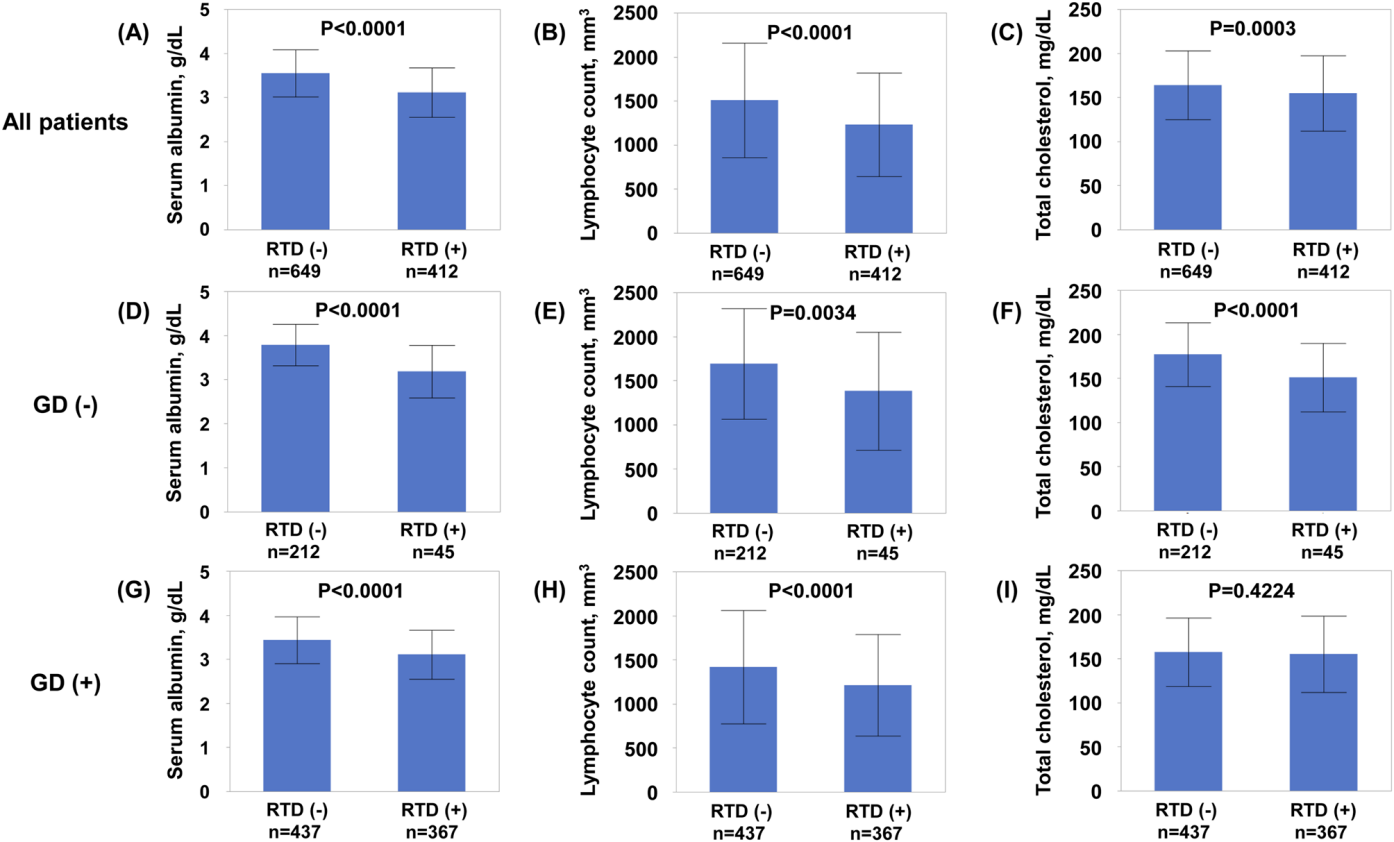
Association between controlling nutritional status score components and RTD in all heart failure patients (**A–C**), in heart failure patients without GD (**D–F**), and in heart failure patients with GD (**G–I**). *GD* glomerular damage, *RTD* renal tubular damage.

**Figure 3:**
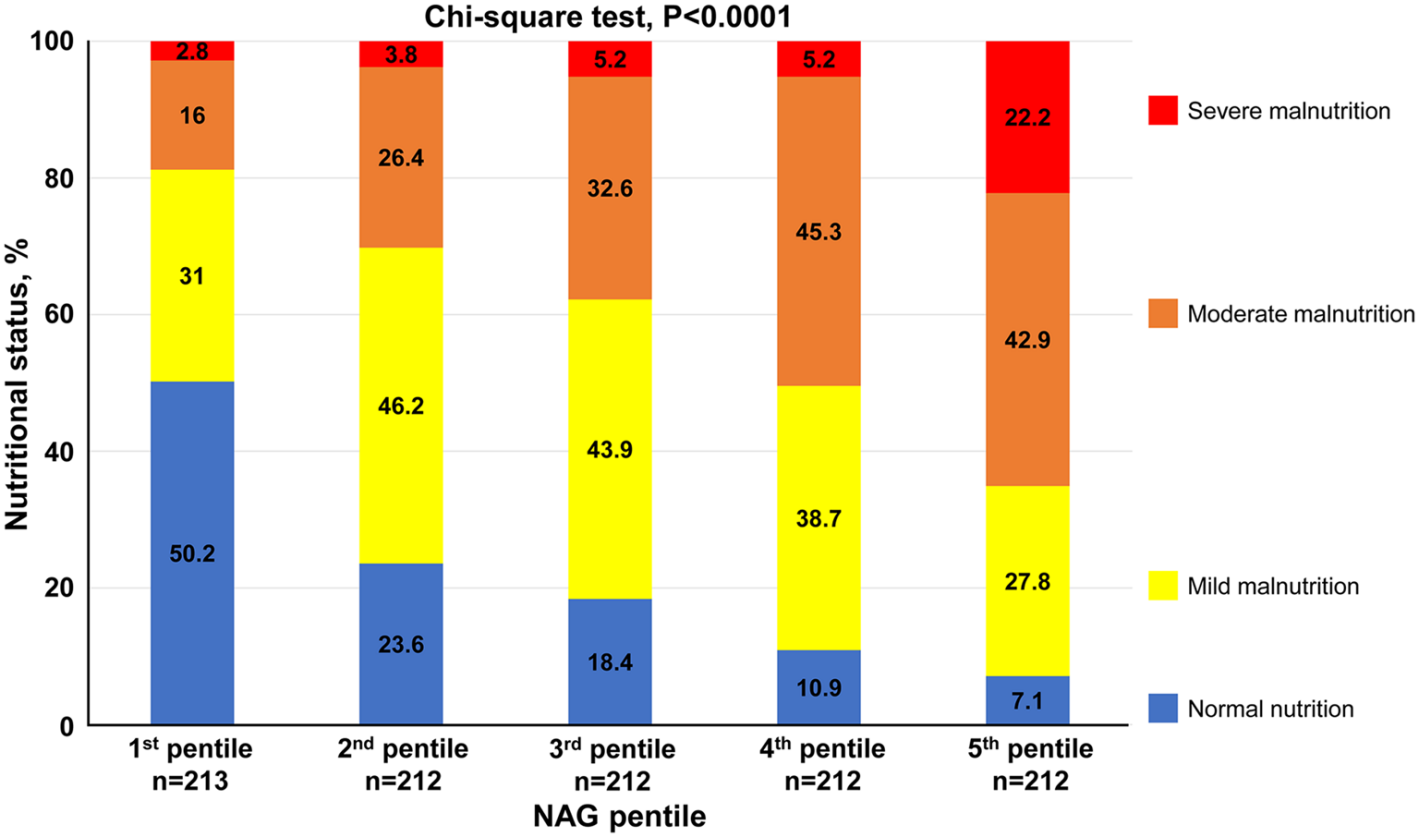
Association between malnutrition severity and renal tubular damage severity.

To examine the association between RTD and CONUT score more precisely, we compared CONUT components between patients with and without RTD. As shown in Fig. 2, patients with RTD had lower serum albumin, lymphocyte count, and total cholesterol than those without RTD in all HF patients. Subgroup analysis in patients without GD showed that patients with RTD also had lower serum albumin, lymphocyte count, and total cholesterol than those without RTD. Similarly, subgroup analysis in patients with GD showed that patients with RTD also had lower serum albumin and lymphocyte counts than those without RTD. To examine the association between RTD and malnutrition more precisely, we divided all patients into pentiles based on the NAG level, since NAG was not normally distributed. As shown in Fig. 3, the frequency of malnutrition increased with advancing RTD.

**Figure 4:**
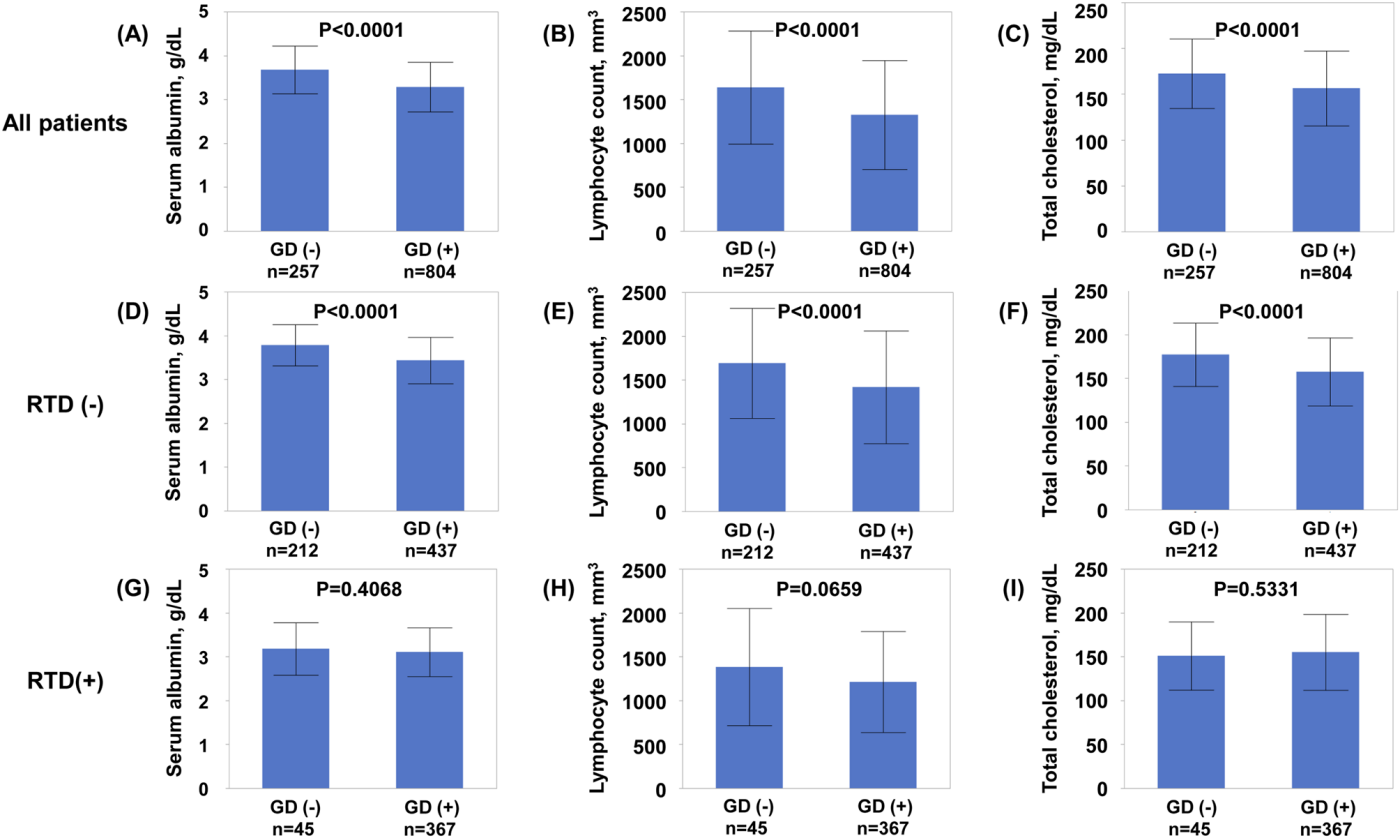
Association between controlling nutritional status score components and GD in all heart failure patients (**A–C**), in heart failure patients without RTD (**D–F**), and in heart failure patients with RTD (**G–I**). *GD* glomerular damage, *RTD* renal tubular damage.

As shown in Fig. 4, patients with GD had lower serum albumin, lymphocyte count, and total cholesterol than those without GD in all patients with HF. Subgroup analysis in patients without RTD showed that patients with GD also had lower serum albumin, lymphocyte count, and total cholesterol than those without GD. However, subgroup analysis in patients with RTD showed no significant differences in serum albumin, lymphocyte count, and total cholesterol between patients with and without GD.

To determine the risk of malnutrition in patients with HF, we performed univariate and multivariate logistic analyses. Univariate analysis showed that age, NYHA functional class, eGFR, BNP, hsCRP, microalbuminuria, reduced eGFR, GD, RTD, left ventricular end diastolic dimension, and diuretics use were related to the presence of malnutrition (Table 2). Multivariate logistic analysis demonstrated that RTD, but not GD, was significantly related to the presence of malnutrition after adjustment for age, NYHA functional class, BNP, hsCRP, left ventricular end diastolic dimension and diuretics use (Table 2).

**Table 2:** Univariable and multivariable logistic analyses for malnutrition defined by high CONUT score.

Variables	Univariable analysis			Multivariable analysis		
	OR	95% CI	P value	OR	95% CI	P value
Age	1.04	1.03–1.06	< 0.0001	1.03	1.01–1.04	< 0.0001
Male vs. female	0.93	0.72–1.19	0.5417			
**NYHA functional class**						
III/IV vs. II	3.36	2.56–4.44	< 0.0001	1.65	1.19–2.30	0.0029
Atrial fibrillation	1.27	0.98–1.64	0.0703			
Hypertension	1.02	0.75–1.39	0.8846			
Diabetes mellitus	1.27	0.99–1.63	0.0627			
Dyslipidemia	1.01	0.79–1.30	0.9123			
eGFR*	0.78	0.68–0.89	0.0001			
BNP*	1.88	1.63–2.19	< 0.0001	1.41	1.17–1.72	0.0003
hsCRP*	2.30	2.01–2.64	< 0.0001	1.82	1.56–2.14	< 0.0001
Microalbuminuria	2.77	2.13–3.61	< 0.0001			
Reduced eGFR	1.94	1.51–2.40	< 0.0001			
GD	3.58	2.58–5.01	< 0.0001	1.23	0.80–1.91	0.3376
RTD	3.40	2.63–4.41	< 0.0001	1.95	1.44–2.66	< 0.0001
LVEDD*	0.88	0.77–0.99	0.0465	0.89	0.76–1.05	0.1650
LVEF*	1.29	0.71–2.37	0.4029			
Diuretics use	1.93	1.49–2.53	< 0.0001	1.78	1.28–2.49	0.0006

*Per 1-SD increase.

*BNP* brain natriuretic peptide, *CI* confidence interval, *CONUT* controlling nutritional status, *eGFR* estimated glomerular filtration rate, *GD* glomerular damage. *OR* odds ratio, *hsCRP* high sensitivity C-reactive protein, *LVEDD* left ventricular end diastolic dimension, *LVEF* left ventricular ejection fraction, *NAG* *N*-acetyl-beta-d-glucosamidase, *NYHA* New York Heart Associations, *RTD* renal tubular damage.

**Figure 5:**
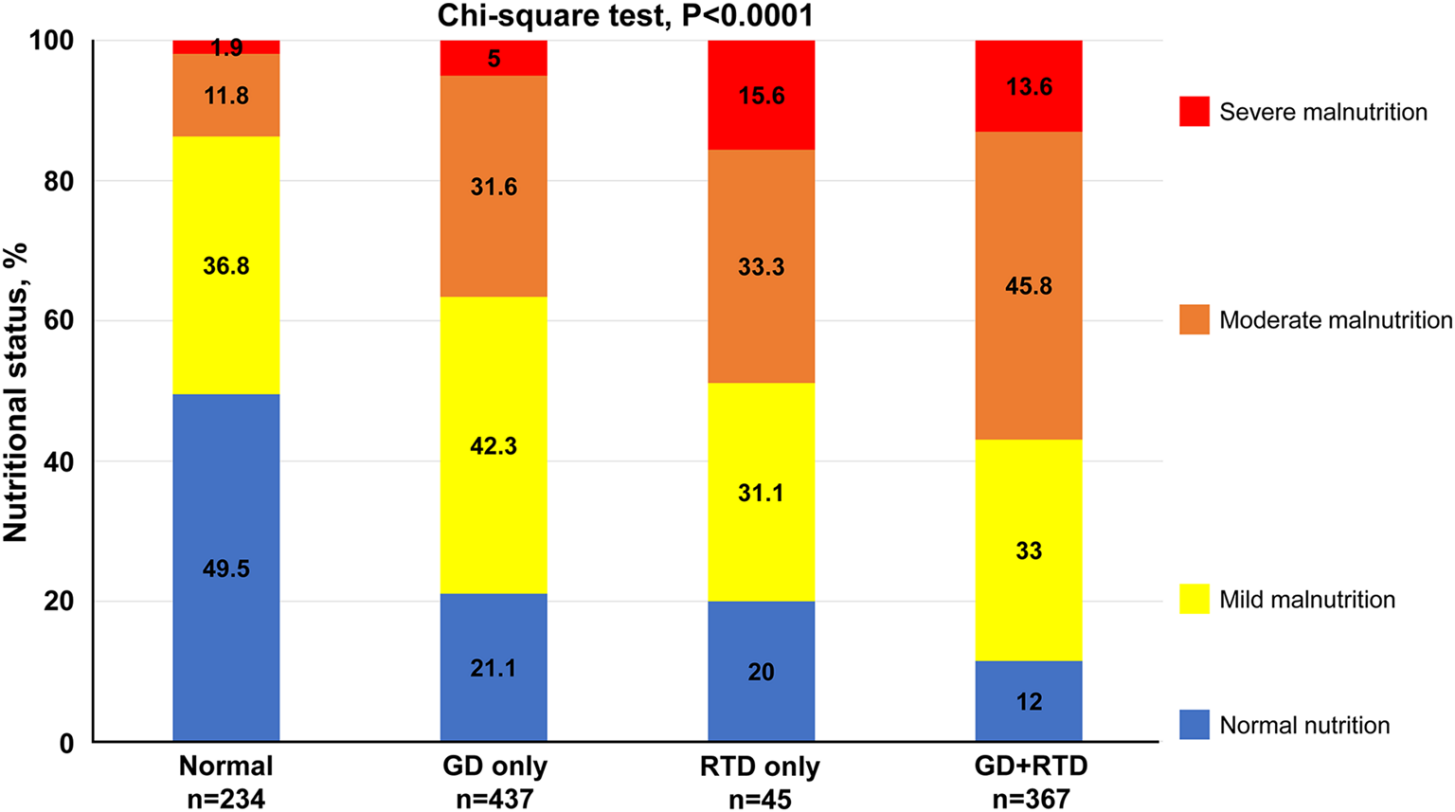
Association between malnutrition severity and type of renal dysfunction. *GD* glomerular damage, *RTD* renal tubular damage.

All patients were divided into four groups based on the presence of GD and RTD. As shown in Fig. 5, the frequency of moderate to severe malnutrition was higher in patients with GD than in those with normal renal function, but it was much higher in patients with RTD and those with GD and RTD. These findings suggest that the frequency of malnutrition differs according to the type of renal dysfunction.

### Clinical outcomes with comorbid malnutrition and renal dysfunction

**Figure 6:**
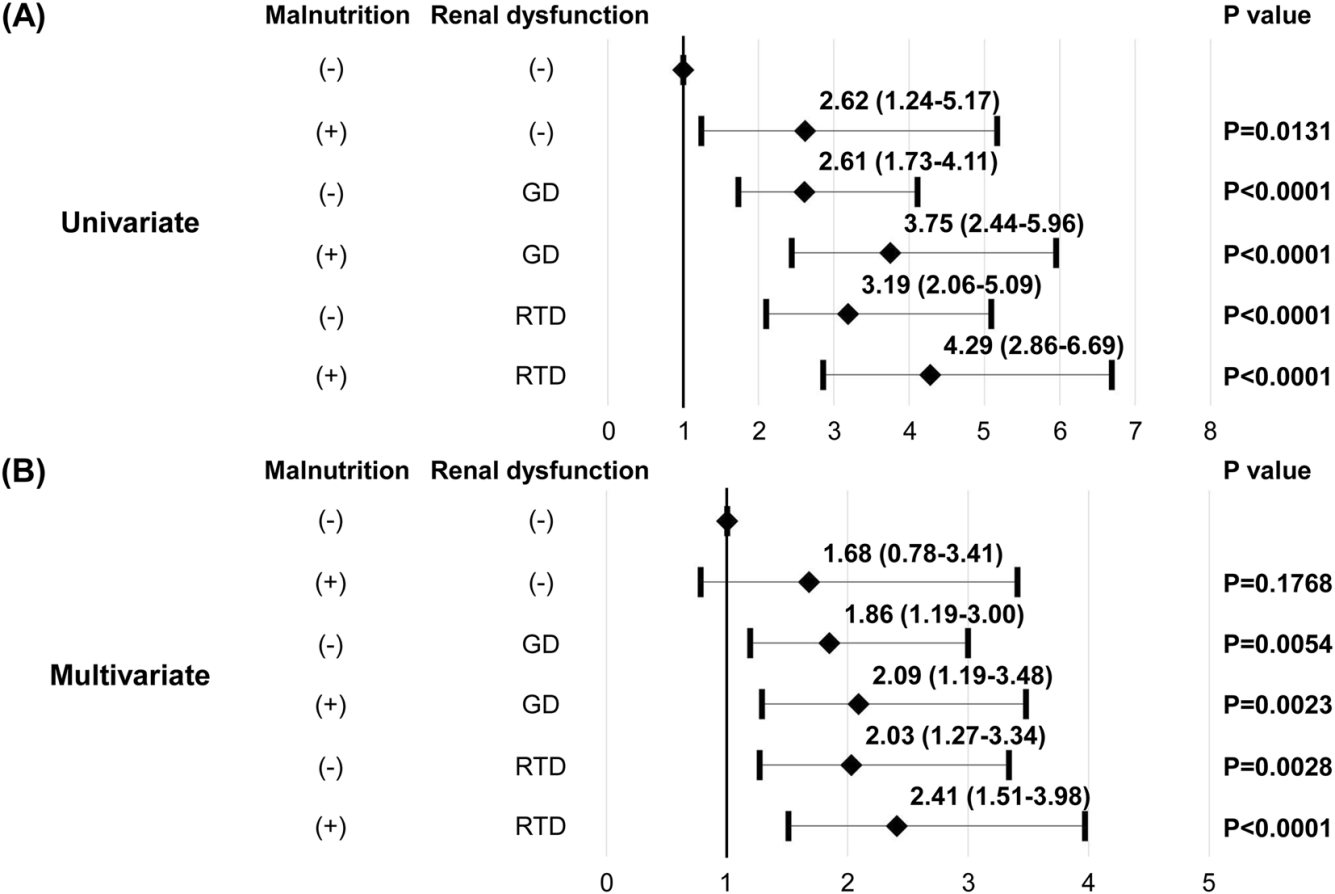
Hazard ratios of malnutrition, GD, malnutrition and GD, RTD, and malnutrition and RTD compared with normal nutritional status and renal function in the univariate (**A**) and multivariate (**B**) Cox proportional hazard regression analysis. Multivariate Cox proportional hazard regression analysis was adjusted for age, sex, NYHA functional class, brain natriuretic peptide, high-sensitivity C-reactive protein and diuretics use. *GD* glomerular damage, *NYHA* New York Heart Association, *RTD* renal tubular damage.

There were 360 cardiac events, including 78 cardiac deaths and 282 HF rehospitalizations, during the study period. To examine the impact of comorbid malnutrition and renal dysfunction on clinical outcomes in patients with HF, we divided all patients into six groups: normal nutritional status and normal renal function, malnutrition and normal renal function, normal nutritional status and GD, malnutrition and GD, normal nutritional status and RTD, and malnutrition and RTD. As shown in Fig. 6A, the univariate Cox proportional hazard regression analysis demonstrated that the hazard ratios for malnutrition and normal renal function, normal nutritional status and GD, malnutrition and GD, normal nutritional status and RTD, and malnutrition and RTD were significantly higher than those in normal nutritional status and normal renal function. A multivariate Cox proportional hazard regression analysis showed that the hazard ratios for normal nutritional status and GD, malnutrition and GD, normal nutritional status and RTD, and malnutrition and RTD were significantly higher than those in normal nutritional status and normal renal function, but not malnutrition and normal renal function (Fig. 6B).

## Discussion

The novel findings from the present study were as follows: (1) most patients with malnutrition showed renal dysfunction such as GD and RTD; (2) patients with renal dysfunction, either GD or RTD, showed impaired nutritional status; (3) the prevalence rate of malnutrition increased with worsening RTD; (4) multivariate logistic analysis demonstrated that RTD, but not GD, was significantly related to the presence of malnutrition; (5) multivariate Cox proportional hazard regression analysis demonstrated that comorbid malnutrition and renal dysfunction were associated with cardiac events in patients with HF.

We showed that most patients with malnutrition had renal dysfunction, and the prevalence of GD and RTD were 88% and 56%, respectively. These findings suggest the importance of renal dysfunction in the management of nutrition in patients with HF. We showed that patients with RTD had higher CONUT score and lower PNI and GNRI than those without RTD, indicating a close relationship between malnutrition and RTD. HF is a hypercatabolic syndrome, and degraded proteins are released into the circulation as amino acids^15^. Renal tubular cells play an important role in the re-absorption of amino acids. We have reported that RTD is related to hypoalbuminemia in patients with HF^16^. In the present study, we showed a close relationship between RTD and CONUT score components. Reductions in serum albumin and lymphocyte count were potentially derived from the loss of amino acids^17^. Taking these findings into consideration, it was plausible that RTD participates in the development of malnutrition in HF patients.

A large number of reports have indicated a close relationship between malnutrition and CKD as assessed by GD. Unexpectedly, GD was not significantly related to malnutrition, although patients with GD showed lower CONUT score components than those without GD in the absence or RTD. Since previous reports did not distinguish between GD and RTD, GD was considered the main target of nutrition. We raised the possibility that RTD plays an important role in the development of malnutrition. However, RTD is part of the final common pathway to end-stage renal disease and worsening GD in patients with HF, suggesting that patients with RTD advanced to RTD and GD^18,19^. Indeed, 89% of patients with RTD had concomitant GD in this study. Therefore, we could not exclude the effect of worsening GD on the development of malnutrition in HF patients with RTD.

Renal dysfunction is a well-established risk factor for cardiovascular disease worldwide. Accumulating evidence indicates that renal dysfunction is closely associated with cardiac function in patients with HF through activation of the renin-angiotensin system, volume expansion, cytokine secretion, sympathetic nerve activation, and anemia^20–23^. Considering the results from the present study, we raised the possibility that renal dysfunction partially worsened clinical outcomes through malnutrition.

Malnutrition augmented the risk of cardiac events in patients with HF and renal dysfunction, although it was not significantly associated with cardiac events in patients with HF and normal renal function. One explanation is that malnutrition in patients with HF is treatable in the absence of renal dysfunction. Furthermore, there was no established diet for malnutrition in patients with renal dysfunction. Optimal nutrients have been discussed^24^ and several studies have demonstrated the usefulness of a very low-protein diet supplemented with amino acids and ketoacids to improve several metabolic abnormalities such as hyperphosphatasemia, metabolic acidosis, hyperparathyroidism, and dyslipidemia in patients with moderate to advanced CKD^25,26^. In addition, this diet was reported to decrease serum indoxyl sulfate levels^27^, which is a powerful predictor of overall and cardiovascular mortality in patients with CKD^28^. These reports suggested the importance of amino acid supplementation in patients with CKD. Since renal tubular cells play an important role in the re-absorption of amino acids, comorbid malnutrition and RTD might have the greatest risk for cardiac events. It appears that RTD requires more careful attention because of the loss of amino acids, and specific diets for heart and kidney disease are also important to prevent advancing renal dysfunction in patients with HF. Collectively, our findings indicate that comorbid malnutrition and renal dysfunction can put patients at high risk for cardiac events and could provide useful clinical information for the assessment and management of malnutrition in patients with HF. Unfortunately, a therapeutic strategy for malnutrition, GD, and RTD in patients with HF was not established. Further studies are required to determine therapeutic strategies for these patients.

There were several limitations in this study. First, although the mean left ventricular ejection fraction in the present study was equivalent to those reported in the Japanese heart failure study and heart failure registry^29^, it was relatively high compared with that reported in Western countries^30^. However, HF with preserved ejection fraction tended to be more strongly associated with renal dysfunction than HF with a reduced ejection fraction^31^. We could not exclude the effect of HF phenotype on the results. Second, since study population was enrolled before angiotensin receptor neprilysin inhibitor and sodium glucose cotransporter 2 inhibitors were clinically applied for HF in Japan, we could not examine the impact of these medicines on malnutrition, renal dysfunction and clinical outcomes. Finally, since there were several markers for RTD in patients with cardio-renal syndrome^32^, further studies including another marker for RTD is required to validate our results.

In conclusion, renal dysfunction, notably RTD, is associated with the presence of malnutrition. Comorbid malnutrition and renal dysfunction are risk factors for poor clinical outcomes in patients with HF, indicating the importance of managing these.

## Methods

### Ethics statement

All procedures performed in this study were undertaken in accordance with the ethical, institutional, and/or national research committee guidelines, and all were in compliance with the 1964 Helsinki declaration and its later amendments, or comparable ethical standards. All patients provided written informed consent to participate in this study. The study was approved by the Institutional Ethics Committee of Yamagata University School of Medicine (Yamagata University, 2020-344).

### Study subjects

This prospective observational study was conducted to elucidate the impact of comorbid malnutrition and renal dysfunction on the clinical outcomes of patients with HF. We included 1,061 patients who were admitted to our hospital for the diagnosis or treatment of acute and chronic exacerbation of HF. The diagnosis of HF was made by two or more cardiologists who used the generally accepted Framingham criteria, including a history of dyspnea and symptomatic exercise intolerance, with signs of peripheral edema or pulmonary congestion, and radiological or echocardiographic evidence of left ventricular enlargement or dysfunction^33^.

Transthoracic echocardiography was performed by physicians or clinical laboratory technologists who were blinded to biochemical data. The diagnoses of hypertension, diabetes mellitus, and hyperlipidemia were established according to medical records or history of medical therapy. The exclusion criteria included acute myocardial infarction within 3 months preceding admission, active hepatic disease, and malignant disease.

Demographic and clinical data including age, sex, New York Heart Association (NYHA) functional class, and medicines at discharge were collected from patients’ medical records and interviews.

### Malnutrition

Objective nutritional assessment was performed using the CONUT score, PNI, and GNRI. The CONUT score was calculated using the levels of serum albumin, total lymphocyte count, and total cholesterol, per a previous report^5^. Patients with CONUT scores of 0–1 are considered to have a normal nutritional status, those with CONUT scores of 2–4, 5–8, and 9–12 are at mild risk, moderate risk, and severe risk of malnutrition, respectively. Malnutrition was defined as a CONUT score of ≥ 5 or moderate to severe malnutrition^6^. PNI and GNRI were calculated using the following equations, respectively: PNI = 10 × serum albumin in g/dL + 0.005 × total lymphocyte count in mm^3^, and GNRI = 14.89 × serum albumin in g/dL + 41.7 × (body weight in kg/ideal body weight in kg)^3,4^.

### Renal function

Urine and venous blood samples were collected in the early morning within 24 h of admission. The *N*-acetyl-β-d-glucosamidase (NAG) level, a marker of early tubulointerstitial damage, was measured from single-spot urine specimens. High NAG was defined as an NAG level > 14.2 U/gCr, according to a previous report^12^. We also quantitatively measured urinary albumin level by immunoturbidimetry in a single-spot urine specimen collected early in the morning. Urinary albumin levels were corrected for urinary creatinine levels to determine the urinary albumin to creatinine ratio (UACR).

The glomerular filtration rate (GFR), a standard indicator of renal function, were estimated using the following equations: eGFR in males = 194 × Cr^−1.094^ × Age^−0.287^, and eGFR in females = (194 × Cr^−1.094^ × Age^−0.287^) × 0.739.

Renal dysfunction was defined as a reduced GFR as assessed by eGFR, the presence of albuminuria as assessed by UACR, and/or high NAG levels (> 14.2 U/gCr). Normal renal function was defined as a preserved eGFR (≥ 60 mL/min/1.73 m^2^), absence of albuminuria, and low NAG levels (≤ 14.2 U/gCr). Glomerular damage was defined as a reduced GFR as assessed by eGFR and/or the presence of albuminuria as assessed by UACR.

There were no missing data at baseline. We included outliers of NAG and UACR by utilizing them as categorical variables.

### Biochemical markers

Blood samples were collected to measure brain natriuretic peptide (BNP) levels. The samples were transferred to chilled tubes containing 4.5 mg ethylenediaminetetraacetic acid disodium salt and aprotinin (500 U/mL) and centrifuged at 1000×*g* for 15 min at 4 °C. The clarified plasma samples were frozen, stored at − 70 °C, and thawed immediately before the assay. BNP concentrations were measured using a commercially available radioimmunoassay specific for human BNP according to manufacturer’s protocol (BNP-JP Abbott, Abbott Japan Co., Ltd., Chiba, Japan). Furthermore, high-sensitivity C-reactive protein (hsCRP) levels were measured at the same time.

### Endpoints and follow-up

Patients were prospectively followed for a median duration of 688 days (interquartile range, 192–1034 days). Patients were followed up using medical records or telephone twice a year until 1500 days after discharge since cardiac events occurs after enrollment. Nine patients were lost to follow-up since they moved or could not be contacted. The endpoints were cardiac events, including progressive HF requiring rehospitalization, and cardiac death, defined as death due to progressive HF or sudden cardiac death. Sudden cardiac death was defined as death without definite premonitory signs or symptoms and was diagnosed by the attending physician or cardiologist after patients were transported to the emergency department. There were 43 non-cardiac deaths during the follow-up period.

### Statistical analysis

Continuous variable normality was checked using the Shapiro–Wilk test prior to analysis. All values are expressed as mean ± SD or median (interquartile range). We performed t-tests and chi-square tests to compare continuous and categorical variables, respectively. Because BNP and hsCRP values were not normally distributed, we used logarithm-transformed BNP and hsCRP values in logistic and Cox proportional hazard regression analyses. The association of the severity of malnutrition with NAG levels and the type of renal dysfunction were analyzed using the chi-square test. Logistic analysis was performed to identify the risk of malnutrition. The selected predictors and established risk factors for malnutrition were entered into a multivariate analysis. Confounder selection was based on the previous paper^34^ (Supplemental Table 1). Cox proportional hazard analysis was performed to examine the impact of comorbid malnutrition and renal dysfunction on cardiac events. Multicollinearity was checked using the variance inflation factor. Significant predictors (p < 0.05) in the univariate Cox proportional hazard regression analysis were screened using the Bayesian method. The selected predictors and established risk factors for cardiac events were entered into the multivariate analysis. Statistical significance was set at p < 0.05. Statistical analyses were performed using standard software packages (JMP version 12; SAS Institute Inc., Cary, North Carolina, USA).

## Supplementary Information

Supplementary Information.

## Data Availability

Because of the sensitive nature of the data collected for this study, requests to access the dataset from qualified researchers trained in human subject confidentiality protocols may be sent to Yamagata University at tewatana@med.id.yamagata-u.ac.jp.
