# Supplementary material for: Association of malnutrition with renal dysfunction and clinical outcome in patients with heart failure
Source: Sci Rep. 2022 Oct 5;12:16673. doi: 10.1038/s41598-022-20985-z (PMC9535020; doi:10.1038/s41598-022-20985-z)
Supplement: Supplementary file 1 — Supplementary Information. [file 41598_2022_20985_MOESM1_ESM.pdf]

Association of Malnutrition with Renal Dysfunction and Clinical Outcome in Patients with Heart Failure

Yoichiro Otaki (MD, PhD)[a], Tetsu Watanabe (MD, PhD)[a], Mari Shimizu[b], Shingo Tachibana (MD)[a], Junya Sato (MD)[a], Yuta Kobayashi (MD)[a], Yuji Saito (MD)[a], Tomonori Aono (MD)[a], Harutoshi Tamura (MD, PhD)[a], Shigehiko Kato (MD, PhD)[a], Satoshi Nishiyama (MD, PhD)[a], Hiroki Takahashi (MD, PhD)[a], Takanori Arimoto (MD, PhD)[a], Masafumi Watanabe (MD, PhD)[a]

[a] Department of Cardiology, Pulmonology, and Nephrology, Yamagata University School of Medicine, Yamagata, Japan

[b] Faculty of Medicine, Yamagata University School of Medicine, Yamagata, Japan

Correspondence: Tetsu Watanabe, MD, PhD; Department of Cardiology, Pulmonology and Nephrology, Yamagata University School of Medicine, 2-2-2 Iida-Nishi, Yamagata, Japan 990-9585.

E-mail: tewatana@med.id.yamagata-u.ac.jp; Phone: +81-23-628-5302; Fax: +81-23-628-5305.

Supplemental table 1. Variance inflation factor for malnutrition and cardiac events

	Variance inflation factor	
	Malnutrition	Cardiac events
Age	1.19	1.17
Male vs. female		1.05
NYHA functional class III/IV vs. II	1.20	1.24
BNP*	1.38	1.34
hsCRP*	1.19	1.32
Malnutrition		1.32
Glomerular damage	1.33	1.32
RTD	1.15	1.18
LVEDD	1.14	
Diuretics	1.09	1.08

BNP, brain natriuretic peptide; hsCRP, high sensitivity C-reactive protein; LVEDD, left ventricular end diastolic dimension; NYHA, New York Heart Associations; RTD, renal tubular damage.

Supplemental figure. Martingale residuals of six groups divided by malnutrition, glomerular damage, and renal tubular damage.

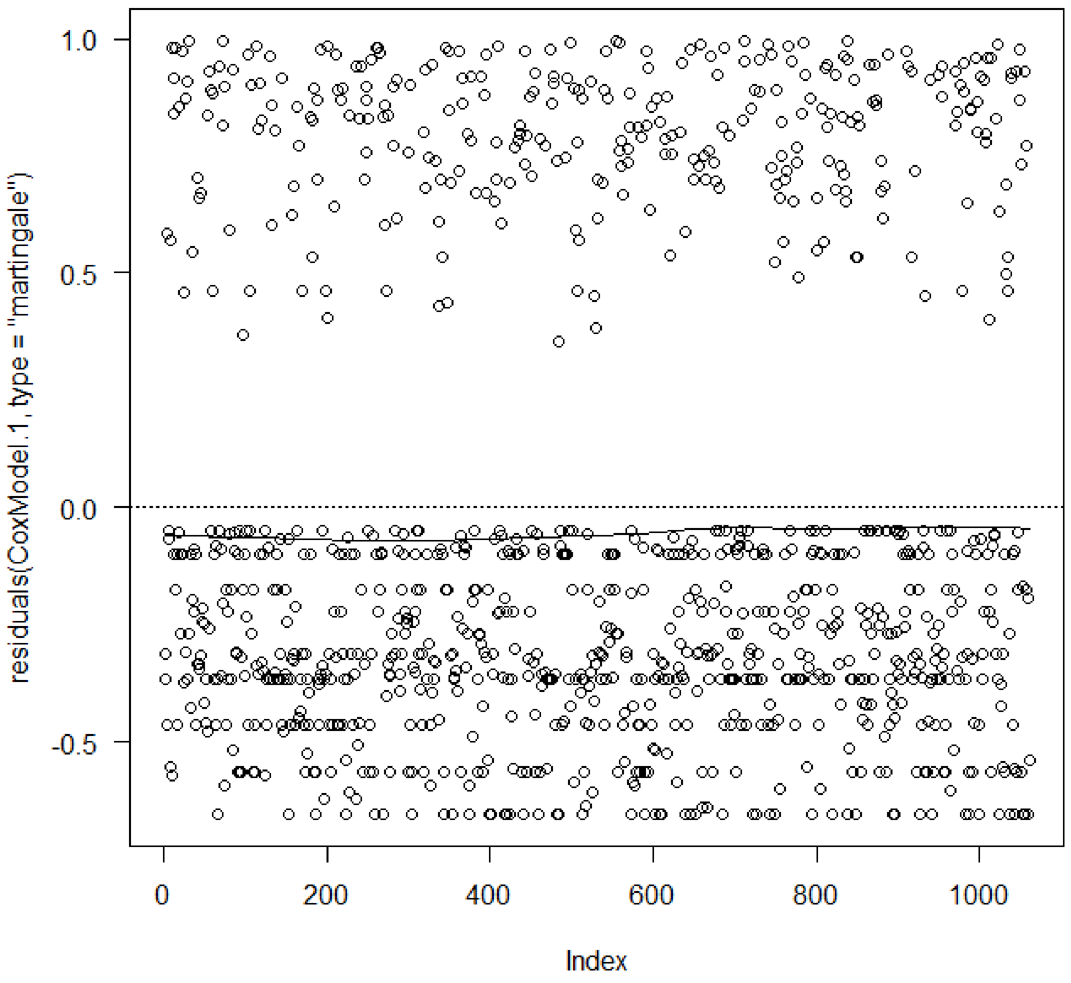

Supplemental table 2. Baseline proportional assumption of six group was calculated by EZR.

print(cox.zph(CoxModel.1))	Chi-square	df	P value
Group	3.6	5	0.61
Global	3.6	5	0.61
